# Multimodal imaging in a child with severe posterior scleritis

**Published:** 2019

**Authors:** Sibel Inan, Elif Ertan, Umit Ubeyt Inan

**Affiliations:** * Department of Ophthalmology, School of Medicine, Health Sciences University, Afyon, Turkey; ** Department of Ophthalmology, Kurtalan State Hospital, Siirt, Turkey; *** Department of Ophthalmology, Parkhayat Hospital, Afyon, Turkey

**Keywords:** posterior scleritis, exudative retinal detachment, multimodal imaging

## Abstract

**Objective:** Posterior scleritis in a child is a rare condition. High-resolution imaging techniques in the course of posterior scleritis have not been published extensively in literature. The authors reported a case of posterior scleritis in a 12-year-old child to demonstrate multimodal imaging techniques in the course of development and improvement of the disease.

**Methods:** Case report that included fundus photography, spectral domain optical coherence tomography with enhanced depth imaging, blue-peak autofluorescence, multicolor imaging, fluorescein angiography, indocyanine green angiography, and ultrasonography.

**Results:** A twelve-year-old healthy boy presented with ocular pain and mild vision loss. His visual acuity was 20/ 32. There was no sign of inflammation on the ocular surface. There were no cells in the anterior chamber or vitreous. Ultrasonography revealed the diagnosis of posterior scleritis. When he was seen the next day for multimodal imaging techniques, he presented with exudative retinal detachment with visual acuity of 20/ 100. One week after the beginning of the therapy, ocular symptoms, and findings resolved and visual acuity improved to 20/ 20.

**Conclusion:** Multimodal imaging techniques, which are important for the diagnosis of posterior scleritis, before and after the treatment, are presented in this case report.

## Introduction

Scleritis is one of the most severe inflammatory disorders affecting the ocular surface. Posterior scleritis is scleral inflammation primarily affecting the scleral-choroidal-retinal layers posterior to equator. Posterior scleritis may involve a localized area (nodular posterior scleritis) or a more common area (diffuse posterior scleritis). In the majority of the cases, posterior scleritis is an autoimmune disease at origin. Sometimes, the diagnosis can be difficult due to variable presentation and ocular findings shared by some other ocular and periocular entities such as Harada’s disease, thyroid orbital inflammatory disease, and optic neuritis. Therefore, the disease can be overlooked or misdiagnosed [**1**]. However, prompt diagnosis and treatment is needed to prevent progression of ocular involvement and subsequent vision loss. We presented the initial and developmental clinical features and characteristics of this condition with multimodal imaging techniques in a child with posterior scleritis. 

## Case report

**Fig. 1:**
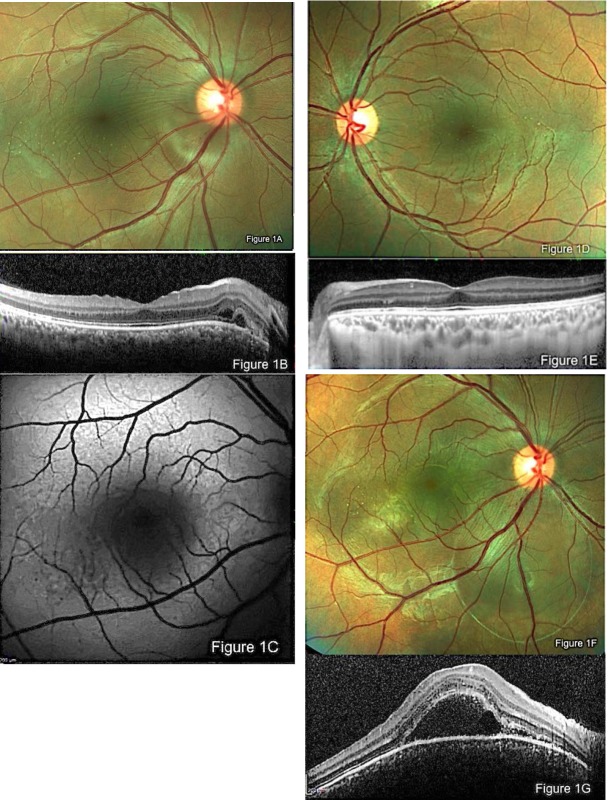
Color photograph of right eye at the presentation. Note radial retinochoroidal striae, tiny retinal deposits, and slight retinal elevation just inferotemporal to the optic disc **(A)**. Some subretinal and intraretinal fluids are seen near the optic disc in OCT (Spectralis OCT; Heidelberg Engineering, Heidelberg, Germany) **(B)**. A granular irregularity of autofluorescence (AF) temporal to the fovea is noted in blue-peak AF **(C)**. Fellow eye shows a normal appearance except for retinal tiny-yellowish deposits in color photograph and a slight increase in choroidal thickness **(D and E)**. Color photograph shows marked elevation of the retina inferior to the disc and macula 7 days after the first presentation **(F)**. OCT shows development of submacular fluid, multiple intraretinal and subretinal hyperreflective dot spots, and convex elevation of RPE-choriocapillaris interface **(G)**

**Fig. 2:**
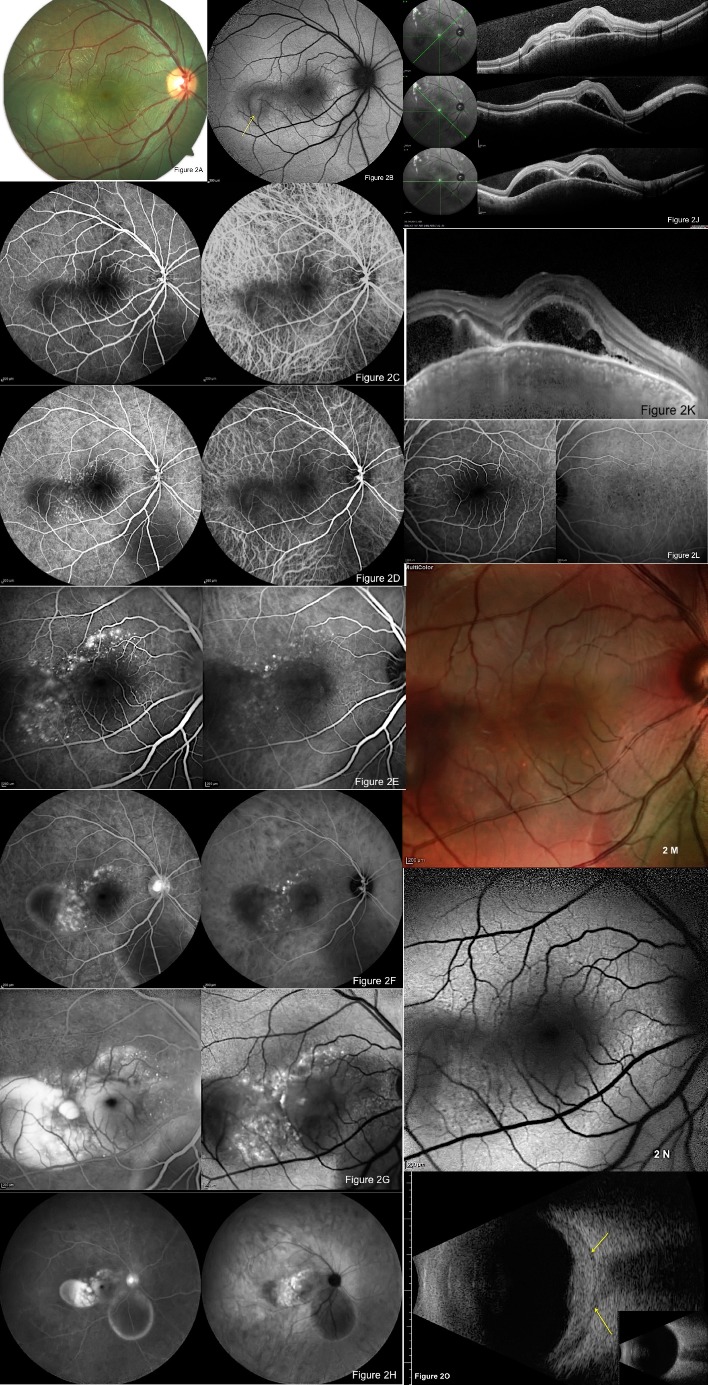
Color photograph the next day shows further elevation in the retina **(A)**. Blue-peak AF shows hypo-AF area suggesting the site of inflammation **(B)**. Early phase of FFA and ICG-A (Spectralis HRA; Heidelberg Engineering, Heidelberg, Germany) also shows a hypo-fluorescence dark area at the presumed site of inflammation **(C)**. Initial-mid phase of FFA and ICG-A show multiple tiny fluorescein leakage spots and enlargement of hypo-fluorescence, respectively **(D)**. Middle-mid phase FFA and ICG-A show enlargement of sites of leakage and appearance of leakage spots, respectively **(E)**. Late-mid phase of FFA and ICG-A show leakage areas confined to the half of the retinal detachment area and enlargement of sites, respectively **(F)**. Late phase of FFA and ICG-A show filling of fluorescence dye and more leakage, respectively **(G)**. Late phase wide-field FFA and ICG-A show complete staining of retinal detachment area in the presumed inflammation site but a hypo-fluorescence in retinal detachment area inferior to the disc **(H)**. Wide field OCT shows multiple detachment areas on different sections **(J)**. EDI-OCT shows enlargement of choroidal layers with elevation of RPE plane possibly due to compression by scleral swelling **(K)**. Middle phase of FFA and ICG-A in the fellow eye show multiple tiny hypo-fluorescent spots **(L)**. Multicolor imaging shows a tiny hypo-pigmentary spot possibly reflecting involvement and leakage of RPE sites **(M)**. Blue-peak AF shows area of inflammatory involvement with hypo-fluorescence and multiple dark spots **(N)**. Ocular ultrasonography (USG, Aviso ultrasound unit, Quantel Medical Systems Inc. Cedex, France) shows marked scleral thickening with T sign and normal ultrasonography in the fellow eye in the smaller picture **(O)**.

**Fig. 3:**
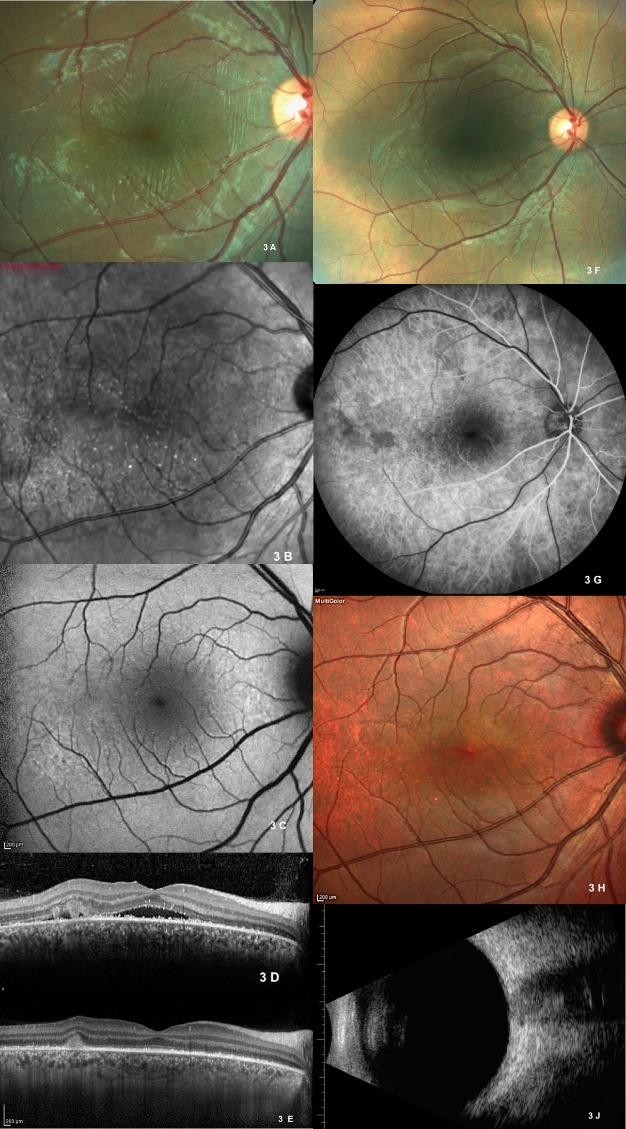
Fig. 3 Color photograph shows resolution subretinal fluid with residual retinal striae 1 week after the initiation of the therapy **(A)**. Infrared reflectance imaging shows multiple dot spots **(B)**. Blue-peak AF shows some hyper-fluorescence granular AF at the site of presumed inflammation **(C)**. OCT shows a decrease in subretinal fluid with residual hyper-reflective material at the site of presumed inflammation and decrease in choroidal thickness **(D)**. OCT shows complete resolution of subretinal fluid with residual hyperreflective subretinal material and residual multiple subretinal hyperreflective dots 2 weeks after the initiation of the therapy **(E)**. Color photograph shows normal retinal appearance but some hypo-pigmentary spots and darker yellow appearance in the temporal to the fovea **(F)**. Early phase of control FFA shows hypo-fluorescent dark spots and a few areas of choroidal filling defects 1 month after the therapy initiation **(G)**. Multicolor imaging shows hypo-pigmentary changes in the original site of the presumed inflammation possibly reflecting RPE involvement at this area **(H)**. OCT shows normal retinal and choroidal configuration at month 1 **(J)**. Control USG shows normalized appearance of scleral wall at month 1.

A twelve-year-old boy with periocular pain and mild visual disturbance in the right eye showed retinal folds in macular area. There was subretinal fluid near the optic disc in optical coherence tomography (OCT). Intraretinal yellowish small deposits in the macular area in both eyes were noted. When the patient was seen in our center seven days after the initial presentation, his vision was 20/32. The anterior sclera was white. There was no cell in the anterior chamber and vitreous. Intraocular pressure was 12 mmHg in both eyes. Retinochoroidal folds radiating from optic nerve head to macular area, retinal elevation in temporal to macula center and inferior to optic disc was observed. Vitreous was also clear. Ocular ultrasonography with a 10 MHz linear-array transducer, revealed the diagnosis of posterior scleritis with increased thickness of choroid and scleral wall and typical appearance of T sign. When the patient came to the department for multimodal imaging the next day, his visual acuity was 20/100 due to the development of serous macular detachment. He also complained of an increase in severity of ocular pain. Selected images from multimodal imaging techniques are shown in **Fig. 1**-**3**.

Systemic evaluation by pediatric rheumatology and work-up performed for investigation of any systemic autoimmune disorder including complete blood cell count, ESR, CRP, RF, ANA, ANCA, anti-dsDNA, Mantoux test, chest X-ray, and syphilis serology revealed no abnormality. 1mg/ kg peroral methylprednisolone, peroral ibufen 30mg/ kg and topical ketorolac tromethamine %0.5 qid, was commenced. The patient’s pain relieved and exudative detachment was resolved five days after the initiation of the treatment. Oral steroid was tapered gradually and then discontinued until 1 month, at which time the clinical picture was resolved completely with an improvement of visual acuity to 20/ 20. The follow-up was usual for 12 months. 

## Discussion

Posterior scleritis is an inflammation of the sclera posterior to the ora serrata and it is usually under-recognized [**1**]. Posterior scleritis usually presents itself as ocular pain, vision loss, refractive changes, proptosis, optic disc swelling, exudative retinal detachment, a bulky appearance reminiscent of choroidal mass, retinal pigment epithelial detachments, choroidal effusion, and chorioretinal folds [**1**-**10**]. Posterior scleritis in children is rarer than in the adult population [**4**]. Fundus fluorescein angiography and optic coherence tomography features have been reported previously. Although the diagnosis can be mistaken with some other diseases, ultrasonographic evaluation helps establishing a definite diagnosis. The most common ultrasound feature is high reflective scleral wall thickening. Increased posterior scleral wall thickness more than 2 mm has been reported in more than half of the patients [**3**]. The other feature is the “T” sign. The “T” sign is seen due to the presence of fluid in the sub-Tenon’s space. The “T” sign was reported in 25% to 41.2% of the cases. Choroidal effusion, localized serous retinal detachment, posterior coats flattening and swelling of the optic disc can also be seen in ultrasonographic imaging [**3**-**6**].

Posterior scleritis is generally autoimmune in origin and it is associated with systemic disease in up to 40% of the patients [**3**]. No systemic abnormality was detected in our case. However, his parents were given an advice to close observation probably for systemic involvement or recurrence in the future. Posterior scleritis can be misdiagnosed if a comprehensive approach with multimodal imaging is not performed. Our case did not have anterior scleritis findings, which was reported one quarter to 3 quarter of the patients [**2**,**3**]. Initially, the only clinical finding observed was retinal striae in the posterior pole. There were no inflammatory signs in anterior or posterior segments. Approximately 25% of the patients have some degree of anterior chamber reaction, and 10% have vitreous cells [**3**].

Serous retinal detachment has been reported as the most frequent clinical sign in both posterior scleritis and Vogt-Koyanagi-Harada disease. Serous retinal detachment seen on OCT, choroidal thickening by EDI-OCT and fluorescein angiographic features implied a diagnosis of Harada disease, but unilateral involvement, absence of cells in the anterior chamber or vitreous and convex elliptic elevation of RPE contour due to compression of choroidal thickness resulting from scleral swelling contrary to diffuse thickening of choroid in Harada disease strongly suggested posterior scleritis. The symptom of periocular pain and ultrasonographic features confirmed the diagnosis. The pain probably results from tendonitis and stretching of Tenon’s capsule by edema into Tenon’s space or stretching or destruction of the nerves that pass through the sclera [**7**]. The clinical picture resolved completely within one month after the beginning of systemic corticosteroid and topical NSAID. Systemic corticosteroid was added to treatment because the majority of cases reported in literature required it for resolution [**3**,**4**,**8**,**10**]. Recurrence was not observed during the 12-month follow-up. However, it has been reported that the majority of recurrences occurred between month 6 and 18 after discontinuation of the therapy [**7**-**10**]. Immunosuppressive therapy may be needed in severe cases associated with systemic diseases and unresponsive to standard treatment. Our case also showed that the immediate diagnosis might be crucial in some patients, because the worsening of the clinical picture and the visual acuity could occur if treatment is delayed even for a few days. 

Increasing awareness of clinical and multimodal imaging features in the course of posterior scleritis could lead to a correct diagnosis with a timely treatment. 
